# The integration of yoga breathing techniques in cognitive behavioral therapy for post-traumatic stress disorder: A pragmatic randomized controlled trial

**DOI:** 10.3389/fpsyt.2023.1101046

**Published:** 2023-04-17

**Authors:** Heidemarie Haller, Dietmar Mitzinger, Holger Cramer

**Affiliations:** ^1^Center for Integrative Medicine and Planetary Health, University Hospital Essen, University of Duisburg-Essen, Essen, Germany; ^2^Insititute of General Practice and Interprofessional Care, University Hospital Tübingen, Tübingen, Germany; ^3^Bosch Health Campus, Stuttgart, Germany

**Keywords:** yoga, pranayama, post-traumatic stress disorder, mental disorder (disease), cognitive behavioral therapy, cbt, randomized controlled trial, safety

## Abstract

**Introduction:**

In trauma-focused Cognitive Behavioral Therapy (TF-CBT), stabilization techniques are used before confrontation ones to increase stress/affect tolerance and thus effectiveness of CBT. This study investigated the effects of pranayama, meditative yoga breathing and breath holding techniques, as a complimentary stabilization technique in patients with post-traumatic stress disorder (PTSD).

**Methods:**

Seventy-four PTSD-patients (84% female, 44.2 ± 13 years) were randomized to receive either pranayama at the beginning of each TF-CBT session or TF-CBT alone. The primary outcome was self-reported PTSD severity after 10 sessions of TF-CBT. Secondary outcomes included quality of life, social participation, anxiety, depression, distress tolerance, emotion regulation, body awareness, breath-holding duration, acute emotional reaction to stress, and adverse events (AEs). Intention-to-treat (ITT) and exploratory per-protocol (PP) analyses of covariance with 95% confidence intervals (CI) were performed.

**Results:**

ITT analyses revealed no significant differences on primary or secondary outcomes, except for breath-holding duration in favor of pranayama-assisted TF-CBT (20.81 s, 95%CI = 13.05|28.60). PP analyses of 31 patients without AEs during pranayama revealed significantly lower PTSD severity (−5.41, 95%CI = -10.17|-0.64) and higher mental quality of life (4.89, 95%CI = 1.38|8.41) than controls. In contrast, patients with AEs during pranayama breath holding reported significantly higher PTSD severity (12.39, 95%CI = 5.08|19.71) than controls. Concurrent somatoform disorders were found to be a significant moderator of change in PTSD severity (*p* = 0.029).

**Conclusion:**

In PTSD patients without concurrent somatoform disorders, the integration of pranayama into TF-CBT might reduce post-traumatic symptoms and increase mental quality of life more efficiently than TF-CBT alone. The results remain preliminary until they can be replicated by ITT analyses.

**Clinical trial registration:**

ClinicalTrials.gov, identifier NCT03748121.

## 1. Introduction

Post-traumatic stress disorder (PTSD) is a result of profound traumatic experiences (1) and is characterized by intrusive memories, avoidance behavior, hyperarousal, dissociation, comorbid affective and somatoform disorders leading to significant psycho-social and economic burden (2). Clinical practice guidelines (3–5) and recent Cochrane reviews (6, 7) recommend cognitive-behavioral approaches focusing on processing traumatic memories as first-line psychotherapeutic treatments for PTSD in adults. However, PTSD other than anxiety disorders is associated with less tolerance to fear-inducing situations, crucial for the alleviation of conditioned anxiety. To increase affect tolerance, Trauma-Focused Cognitive Behavioral Therapy (TF-CBT) uses techniques of stabilization before confrontation (8, 9).

Yoga showed preliminary evidence as a complimentary treatment for PTSD (10–12). To avoid potentially aversive triggering stimuli, studies combined yoga with common stabilization techniques (13) or used trauma-sensitive yoga approaches (14, 15). In contrast to asanas (yoga postures), the effects of pranayama (breathing techniques) are understudied. In healthy adults and different patient samples, breathing interventions are shown to foster emotion regulation and normalize autonomic nervous system (ANS) activity (16–19). In PTSD, initial studies investigated meditative breathing techniques for stabilization (20) that contributed to reduced PTSD severity (21, 22), while breath holding tasks are commonly used as a measure of distress tolerance (23).

This study operationalized pranayama as a combination of mindful breathing techniques for stabilization and breath-holding techniques for intentional inhibition of dorsal-vagal reflexes of the ANS. With pranayama, patients may be able to enhance: (i) the adaptability of their ANS toward a more stabilized ventral-vagal activation (19, 24–26) and (ii) the connectivity of their central executive network with brainstem and amygdala for more intentional control and the experience of self-efficacy (27, 28). This is aimed to increase affect tolerance and improve response to CBT confrontation techniques.

## 2. Materials and methods

### 2.1. Trial design and registration

This study was designed according to CONSORT (29) using a pragmatic design (30). After confirming eligibility, obtaining written informed consent and assessing baseline data, PTSD patients were randomized to either the experimental group receiving pranayama as part of their standard out-patient TF-CBT or the control group receiving standard out-patient TF-CBT alone. Patient-reported outcomes were collected after 10 sessions of TF-CBT. Subsequently, control patients were offered to learn pranayama as well. The trial was conducted between February 2018 and July 2020 at German psychotherapeutic out-patient clinics by 14 psychotherapists specialized in TF-CBT. Before patient recruitment, the trial protocol was approved by the ethics committee of the University of Duisburg-Essen, Germany (17-7703-BO) and registered at ClinicalTrials.gov (NCT03748121).

### 2.2. Randomization

Patients were allocated to groups using block randomization with randomly varying block lengths stratified by therapists. The random sequences were generated by the study biometrician who was not involved in patient recruitment using an online randomization program^1^. The biometrician prepared sealed and opaque envelops, which were opened by the respective study therapist in ascending order of randomization.

### 2.3. Blinding

Patients and therapists could not be blinded to group allocation. Outcome assessment was performed by blinded onsite staff who was not aware of the patient’s group assignment. In cases where therapists did not employ an assistant who could be blinded, patients returned questionnaires in sealed envelopes.

### 2.4. Sample criteria

Inclusion criteria were an age between 18 and 70 years, an ICD-10 F43.1 diagnosis with a severity of at least 33 points on the Post-traumatic Stress Disorder Checklist (PCL-5) (31, 32), and receiving out-patient TF-CBT. Exclusion criteria were severe comorbid mental or somatic diseases that did not allow the patient to perform pranayama, pregnancy, and a regular practice of yoga or Pilates in the last 12 months.

Sample size estimations were calculated considering the effects of yoga on PTSD severity measured by the PCL using G*Power software (release 3.1.9.4, Kiel University, Germany) (33). A meta-analysis (34) showed a mean standardized effect of yoga versus waiting list of −0.75. To detect this group difference with a power of 0.80, a two-sided t-test with α-level of 0.05 requires 58 patients. Based on previous studies that resulted in dropout rates of up to 20% (35), a final sample size of 74 patients was calculated.

### 2.5. Study interventions

In the experimental group, three rounds of pranayama lasting 5–10 min were performed at the beginning of each TF-CBT session. Each round started with 1 min of mindful stabilization exercise followed by an intentional breath-holding exercise. Therapists were instructed to select one stabilization exercise starting with Kapalabhati (passive inspiration with fast forceful expiration). In cases where Kapalabhati was not tolerable for the patient, Ujjayi (slow, deep breathing with contraction of the trachea) or, if Ujjayi was not tolerable, Nadi Shodhana (slow, alternate nostril breathing) should be performed. Consecutively, breath-holding (Kumbhaka) was executed. Kumbhaka was physically supported by Jalandhara-Bandha, a specific muscle contraction technique of the throat that facilitates holding of the breath after complete inhalation. The exercise ended with exhalation, when the individual reflex prevailed over volition (36, 37). After the three rounds of pranayama, standard guideline TF-CBT was performed for the remaining time of 40 to 45 min.

In the control group, the intervention consisted of 50 min of standard guideline TF-CBT (5). Stabilization techniques included trauma education, progressive muscle relaxation, self-control and affect-control methods, imagination, and self-care/resource development and were followed by standard confrontation techniques (5).

### 2.6. Outcome measures

The primary outcome was PTSD severity on the 20-item PCL-5 scale (31, 32) after 10 sessions of TF-CBT. Scores range from 0 to 80 points with higher ones representing higher symptom severity. The cut-off for clinically relevant symptom severity is 33 points. A reduction of 5 points is reported as the minimal threshold for treatment response (38).

Secondary outcomes included: the Short Form 12 Health Survey (SF-12) (39), the Patient-Reported Outcomes Measurement Information System (PROMIS) Ability to Participate in Social Roles and Activities Scale (40), the Beck Anxiety Inventory (BAI) (41, 42), the Beck Depression Inventory Revision (BDI-II) (43), the Distress Tolerance Scale (DTS) (44), the Emotion Regulation Scale (ERQ) (45, 46), the Scale of Body Connection (SBC) (47), the Emotional Stress Reaction Questionnaire (ESRQ) (48), and the Breath-Holding Task (BHT). For the BHT, patients were instructed to breath normally while sitting relaxed on a chair, then inhale and hold their breath as long as possible (23). The BHT-1 time indicates the threshold of the respiratory reflex, assessed when the patient lifted a finger. The BHT-2 time indicates the duration of the intentional suppression of the respiratory reflex, assessed at exhalation. BHT-1 plus BHT-2 resulted in the BHT total time. Equivalent to previous studies, the average duration (in seconds) across three consecutive rounds was calculated (49).

Safety was operationalized as the number of patients with adverse events (AE) or study withdrawals due to AEs. AEs were defined as any untoward medical occurrence in a patient, which did not have to have a defined causal relationship with the treatment being studied. Cases of any untoward medical occurrence that, at any dose, resulted in death, were life-threatening, required inpatient hospitalization, or caused persistent or significant disability were assessed as serious AEs (50).

While all outcomes were measured at baseline and after 10 sessions of TF-CBT, the ESRQ and safety were measured before and after each therapy session as well. Finally, the expectation subscale of the Treatment Credibility Scale was given at baseline to measure treatment expectation on a numeric rating scale (NRS) (51).

### 2.7. Statistical analysis

Confirmatory analysis of the primary outcome was based on the intention-to-treat (ITT) population including all randomized patients, regardless of missing data or whether or not the patient fully adhered to the protocol. Independent t-tests and chi-square tests were used to analyze whether missing data are completely at random (MCAR). MCAR data were imputed 25 times using fully conditional specification iterations (52). Considering an α-level of 0.05, the primary outcome was analyzed as a function of treatment group and patients’ expectations and respective baseline values (linear covariates) using univariate ANCOVA with post-hoc two-sided t-tests. Results were displayed as mean differences (MD) between the groups and 95%CIs.

For exploratory analyzes of secondary outcomes, comparable ANCOVA models were applied to both the ITT population as well as the per-protocol (PP) population, consisting of all ITT patients but differentiating between those who were able to perform pranayama breath and breath holding techniques as instructed and those who were not because of reporting AEs. AEs were analyzed descriptively and by repeated-measures ANCOVAs with patients’ expectations as a linear covariate.

(Non-)linear regression analyzes were executed to analyze, whether the performance of a specific meditative pranayama exercise (Kapalabhati, Ujjayi, or Nadi Shodhana) did influence change in PTSD severity.

Finally, moderation analyzes were executed to identify variables of patients (W) such as sociodemographics, disease characteristics, or comorbidities that moderate the effect of treatment allocation (X) on change in PTSD severity (Y) using PROCESS (release 2.5.2) macro for SPSS with bootstrapping of 5,000 samples and 95%Cis. PROCESS tests Y as a linear function of: Ŷ = constant+(b_1_ + b_3_W)X + b_2_W. If the effect of X on Y varies with W, b_3_ has to be significantly different from zero (53).

All analyzes were performed using SPSS software (release 27.0, IBM, United States).

## 3. Results

### 3.1. Patient flow

**Figure 1:**
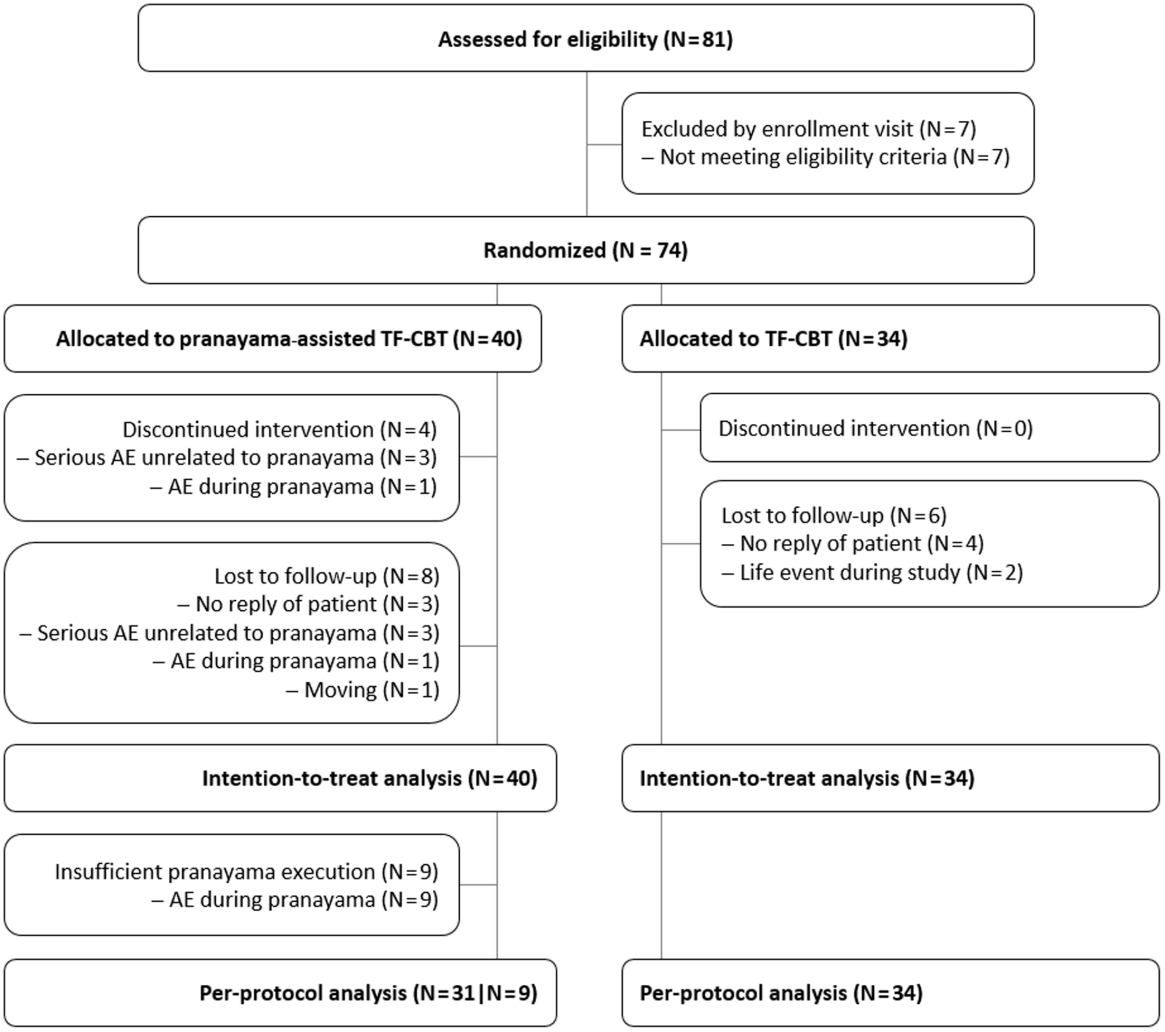
CONSORT patient flow. *N* = Number of patients, TF-CBT = Trauma-focused cognitive behavioral therapy.

We screened 81 patients, of which seven were excluded because of PCL-5 scores below 33 (Figure 1). Of the 74 randomized patients, four discontinued pranayama and were lost to follow-up because of one non-serious AE during pranayama breath holding exercise (flashback of suffocation) and three serious AEs unrelated to pranayama (Table 1). Further four pranayama patients dropped-out because of relocation and unknown reasons. In total, nine pranayama patients reported 20 minor but recurrent AEs during or subsequent to pranayama causing insufficient execution of pranayama because of emerging anxiety and/or feelings of constriction in eight cases and, as mentioned above, dropout in one case. Further three pranayama patients reported six minor and temporary AEs during or subsequent to pranayama, not influencing pranayama execution or study participation. In the control group, data of six patients were lost to follow-up as a result of life events (financial problems and work accident) and unknown reasons. One additional patient of the control group reported one minor AE not causing dropout.

Further safety analyzes of patients’ acute emotional reactions to stress revealed that ITT patients of the pranayama group reported significantly higher predominance of positive emotions on the ESRQ directly after each pranayama performance as compared to control before each session (MD = 2.17, 95%CI = 0.45|3.89; *p* = 0.014). ESRQ ratings assessed after each TF-CBT session did not significantly differ between ITT groups (*p* ≥ 0.050).

**Table 1:** Number of adverse events.

	Pranayama-assisted TF-CBT (*N* = 15)	TF-CBT (*N* = 1)
AE during or subsequent to pranayama (*n*)		
Anxiety	6	0
Breathlessness	4	0
Cough	1	0
Dizziness	6	0
Feeling of constriction	4	0
Flashback of suffocation	1	0
Headache	2	0
Neck pain	2	0
AE unrelated to pranayama (*n*)		
Death (congenital heart defect)	1	0
Hospitalization (orofacial cleft surgery|stent implantation)	2	0
Fatigue	0	1

AE = Adverse Event, *N* = Number of patients with adverse events, *n* = Number of adverse events, TF-CBT = Trauma-focused cognitive behavioral therapy.

**Table 2:** Sample characteristics at baseline.

	Pranayama-assisted TF-CBT (*N* = 40)	TF-CBT (*N* = 34)	Total (*N* = 74)
Social demographics			
Age (mean ± SD)	44.18 ± 12.81	44.18 ± 13.45	44.18 ± 13.02
Gender: female|male (N)	34|6	28|6	62|12
BMI: kg/m2 (mean ± SD)	24.39 ± 4.83	25.97 ± 5.39	25.12 ± 5.12
Country of birth: Germany|others (N)	36|4	31|3	67|7
Education: <high school|high school|university (N)	25|4|11	21|3|10	46|7|21
Employment: unemployed|sick leave|employed|retired (N)	5|7|19|9	6|4|19|5	11|11|38|14
Marital status: relationship|no relationship (N)	21|19	25|9	46|28
Health insurance: public|private (N)	37|3	31|3	68|6
Treatment expectation (NRS) (mean ± SD)	6.61 ± 1.73	6.97 ± 2.05	6.77 ± 1.88
PTSD characteristics			
DSM-5 criterion A: LEC-5 PTSD|complex PTSD (N)	3|37	11|23	14|60
DSM-5 criterion B: met|not met (PCL-5 intrusion) (N)	26|14	23|11	49|25
DSM-5 criterion C: met|not met (PCL-5 avoidance) (N)	28|12	22|12	50|24
DSM-5 criterion D: met|not met (PCL-5 cognition/mood) (N)	20|20	19|15	39|35
DSM-5 criterion E: met|not met (PCL-5 arousal) (N)	18|22	16|18	34|40
PTSD severity (PCL-5) (mean ± SD)	44.39 ± 14.96	42.78 ± 13.25	43.65 ± 14.13
Years since trauma exposure (mean ± SD)	21.66 ± 18.91	17.38 ± 16.83	19.55 ± 17.91
Somatoform disorder: met|not met ICD-10 F45	12|28	11|23	23|51

DSM-5 = Diagnostic and Statistical Manual of Mental Disorders-Fifth Edition, LEC-5 = Life Events Checklist for DSM-5, N = Number of patients, NRS = Numeric Rating Scale, PCL-5 = Posttraumatic Stress Disorder Checklist for DSM-5, PTSD = Post-Traumatic Stress Disorder, SD = Standard deviation, TF-CBT = Trauma-focused Cognitive Behavioral Therapy.

### 3.2. Baseline characteristics

Patients’ age at baseline ranged from 18 to 67 with a mean of 44.18 ± 13.02 years. Most of the patients were female (83.78%) and were born in Germany (90.54%). Levels of education below high school (62.16%) prevailed, followed by patients with university degree (28.38%) and those with high school graduation (9.46%). Half of the sample was employed (51.35%). Treatment expectations were on average 6.77 ± 1.88 NRS. Patients all had an ICD-10 F43.1 diagnosis with 81.08% reported complex DSM-5 PTSD and 18.92% PTSD of a moderate severity of on average 43.65 ± 14.13. In addition, 31.08% of the patients were diagnosed with an ICD-10 F45 somatoform disorder. Trauma exposure was 19.55 ± 17.91 years ago (Table 2).

No baseline differences were found comparing study adherers and non-adherers (Table 3). In the experimental group, analyzes of patients performing pranayama without AEs versus not yielded the same results with one exception: ICD-10 F45 somatoform disorders were significantly more prevalent in patients who experienced AEs during or subsequent to pranayama (*p* < 0.001; Table 3).

**Table 3:** Dropout analyzes.

	Completed the study (*N* = 60)	Lost to follow-up (*N* = 14)	*P*	Execution of pranayama without AEs (*N* = 31)	Execution of pranayama with recurrent AEs (*N* = 9)	*P*
Age (mean ± SD)	44.52 ± 12.91	42.71 ± 13.84	0.644	43.61 ± 13.25	46.11 ± 11.65	0.613
Gender: female|male (*N*)	51|9	11|3	0.687	27|4	7|2	0.602
Education: <high school|high school|university (*N*)	37|7|16	9|0|5	0.373	20|4|7	5|0|4	0.292
PTSD severity at baseline (PCL-5) (mean ± SD)	43.42 ± 12.65	44.64 ± 19.80	0.828	44.43 ± 15.79	44.22 ± 12.49	0.971
Somatoform disorder: met|not met ICD-10 F45	19|41	4|10	0.548	3|28	9|0	<0.001
Anxiety at baseline (BAI) (mean ± SD)	28.17 ± 10.49	28.63 ± 14.15	0.890	28.97 ± 12.08	30.41 ± 13.42	0.761
Depression at baseline (BDI-II) (mean ± SD)	23.33 ± 10.06	27.49 ± 13.75	0.200	25.16 ± 12.01	28.34 ± 9.69	0.465
Distress tolerance at baseline (DTS) (mean ± SD)	3.11 ± 0.71	3.16 ± 1.06	0.870	3.18 ± 0.71	3.03 ± 0.75	0.572
Treatment expectation (NRS) (mean ± SD)	6.75 ± 1.67	6.8.6 ± 2.66	0.892	6.86 ± 1.75	5.78 ± 1.48	0.101
Pranayama (Kapalabhati | Ujjayi | Nadi Shodhana) (N)	7|8|11	0|2|0	0.175	4|8|7	3|1|5	0.346

AE = Adverse Event, BAI = Beck Anxiety Inventory, BDI = Beck Depression Inventory, DTS = Distress Tolerance Scale, *N* = Number of patients, NRS = Numeric Rating Scale, PCL-5 = Posttraumatic Stress Disorder Checklist for DSM-V, SD = Standard deviation.

### 3.3. Analyzes of PTSD severity

ITT and PP results are shown in Tables 4, 5. While both groups improved over time, the primary ITT analysis revealed no statistically significant different post-treatment PCL-5 scores between groups (*p* = 0.551). However, when differentiating between patients who reported recurrent AEs during pranayama with and those who were able to perform pranayama without AEs, exploratory PP analyzes showed a statistically significant difference in PTSD severity when compared to control (*p* < 0.001). *Post hoc* tests revealed a PCL-5 decrease of −5.41 points (95%CI = -10.17|-0.64, *p* = 0.027) in the pranayama group without recurrent AEs and an increase of 12.39 points (95%CI = 5.08|19.71, *p* = 0.001) in patients who were unable to cope with AEs during pranayama in comparison to control patients.

**Table 4:** Results (means and standard deviations) of the intention-to-treat analyzes.

	Pranayama assisted TF-CBT (*N* = 40)		TF-CBT (*N* = 34)		Between-group differences (95% CI)	*p*
	Baseline	Follow-up	Baseline	Follow-up		
PTSD severity (PCL-5)	44.39 ± 14.96	35.51 ± 17.06	42.78 ± 13.25	35.30 ± 16.24	−1.55 (−6.69|3.59)	0.551
Physical quality of life (SF-12)	36.81 ± 9.43	39.41 ± 8.73	41.29 ± 11.41	41.40 ± 10.61	0.24 (−3.6|3.84)	0.896
Mental quality of life (SF-12)	33.03 ± 9.25	36.37 ± 8.52	32.10 ± 9.00	33.35 ± 9.39	2.72 (−0.88|6.32)	0.137
Social participation (PROMIS)	40.79 ± 6.37	42.15 ± 5.18	41.23 ± 7.16	42.21 ± 6.73	0.19 (−2.08|2.46)	0.868
Anxiety (BAI)	29.29 ± 12.23	24.75 ± 11.50	26.75 ± 9.73	22.66 ± 9.71	0.44 (−3.45|4.33)	0.823
Depression (BDI-II)	25.88 ± 11.32	22.65 ± 11.64	22.05 ± 10.08	19.55 ± 10.88	−0.27 (−3.24|2.69)	0.854
Distress tolerance (DTS)	3.14 ± 0.71	3.24 ± 0.64	3.06 ± 0.82	3.17 ± 0.71	0.03 (−0.22|0.29)	0.810
Emotion reappraisal (ERQ)	3.77 ± 1.13	4.01 ± 1.17	3.70 ± 1.14	3.91 ± 1.26	0.08 (−0.31|0.46)	0.697
Emotion suppression (ERQ)	4.21 ± 1.21	4.16 ± 1.17	3.74 ± 1.08	4.17 ± 1.32	−0.37 (−0.83|0.01)	0.118
Body awareness (SBC)	2.27 ± 0.51	2.25 ± 0.62	2.26 ± 0.58	2.30 ± 0.63	−0.06 (−0.22|0.09)	0.421
Emotional stress reaction (ESRQ)	1.18 ± 6.44	3.97 ± 5.99	0.14 ± 5.24	2.33 ± 5.48	1.05 (−1.25|3.35)	0.366
Breath-holding duration (BHT)	42.18 ± 22.07	60.70 ± 29.30	37.16 ± 25.50	36.01 ± 19. 76	20.81 (13.05|28.60)	0.000
Breath reflex threshold (BHT-1)	27.68 ± 16.64	39.55 ± 22.47	20.53 ± 9.68	22.10 ± 10.04	11.49 (5.00|18.00)	0.001
Breath reflex suppression (BHT-2)	14.50 ± 13.64	21.15 ± 20.07	16.57 ± 20.67	13.91 ± 13.95	8.80 (3.27|14.32)	0.002

CI = Confidence Interval, BAI = Beck Anxiety Inventory, BDI-II = Beck Depression Inventory Revision, BHT = Breath-Holding Scale, DTS = Distress Tolerance Scale, ERQ = Emotion Regulation Scale, ESRQ = Emotional Stress Reaction Questionnaire, N = Number of patients, PCL-5 = Post-Traumatic Stress Disorder Checklist for DSM-5, PROMIS = Patient-Reported Outcomes Measurement Information System, PTSD = Post-Traumatic Stress Disorder, SBC = Scale of Body Connection, SF-12 = Short form 12 health survey, TF-CBT = Trauma-focused Cognitive Behavioral Therapy.

**Table 5:** Results (means and standard deviations) of the per-protocol analyzes.

	Pranayama assisted TF-CBT without AEs (*N* = 31)		Pranayama assisted TF-CBT with recurrent AEs (*N* = 9)		TF-CBT (*N* = 34)		*p*	Post-hoc between-group differences (95% CI)		*p*	
	Baseline	Follow-up	Baseline	Follow-up	Baseline	Follow-up		1–3	2–3	1–3	2–3
PTSD severity (PCL-5)	44.43 ± 15.79	31.37 ± 16.12	44.22 ± 12.50	49.79 ± 12.16	42.78 ± 13.25	35.30 ± 16.24	0.000	−5.41 (−10.17|-0.64)	12.39 (5.08|19.71)	0.027	0.001
Physical quality of life (SF-12)	36.61 ± 9.15	40.45 ± 1.39	37.50 ± 10.84	39.48 ± 2.55	41.23 ± 11.41	40.00 ± 1.31	0.938	0.45 (−3.39|4.30)	−0.52 (−6.29|5.26)	0.815	0.859
Mental quality of life (SF-12)	33.25 ± 9.04	38.79 ± 7.58	32.26 ± 10.48	28.04 ± 6.11	32.10 ± 8.99	33. 35 ± 9.39	0.001	4.89 (1.38|8.41)	−5.18 (−10.5|0.22)	0.007	0.060
Social participation (PROMIS)	40.94 ± 6.28	42.60 ± 5.14	40.27 ± 7.02	40.57 ± 5.30	41.23 ± 7.16	42.21 ± 6.73	0.670	0.54 (−1.86|2.95)	−1.11 (−4.81|2.6)	0.654	0.553
Anxiety (BAI)	28.97 ± 12.08	22.79 ± 11.55	30.41 ± 13.41	31.51 ± 8.87	26.75 ± 9.73	22.66 ± 9.71	0.048	−1.22 (−5.20|2.76)	6.50 (0.40|12.61)	0.542	0.037
Depression (BDI-II)	25.16 ± 12.01	20.59 ± 11.27	28.34 ± 8.70	29.75 ± 10.55	22.05 ± 10.08	19.55 ± 10.89	0.023	−1.64 (−4.63|1.35)	4.85 (0.25|9.45)	0.277	0.039
Distress tolerance (DTS)	3.18 ± 0.71	3.29 ± 0.60	3.03 ± 0.75	3.09 ± 0.76	3.06 ± 0.82	3.17 ± 0.71	0.886	0.05 (−0.22|0.32)	−0.04 (−0.46|0.38)	0.712	0.849
Emotion reappraisal (ERQ)	3.91 ± 1.17	4.18 ± 1.11	3.30 ± 0.91	3.44 ± 1.26	3.70 ± 1.14	3.91 ± 1.26	0.805	0.12 (−0.23|0.53)	−0.06 (−0.70|0.58)	0.585	0.855
Emotion suppression (ERQ)	4.24 ± 1.19	4.07 ± 1.06	4.11 ± 1.34	4.47 ± 1.50	3.74 ± 1.08	4.17 ± 1.32	0.219	−0.43 (−0.92|0.06)	−0.14 (−0.88|0.60)	0.085	0.709
Body awareness (SBC)	2.32 ± 0.56	2.33 ± 0.63	2.10 ± 0.23	1.97 ± 0.54	2.26 ± 0.57	2.30 ± 0.63	0.332	−0.03 (−0.20|0.14)	−0.19 (−0.45|0.07)	0.734	0.140
Emotional stress reaction (ESRQ)	1.97 ± 6.34	5.44 ± 5.07	−1.5 6 ± 6.39	−1.13 ± 6.36	0.14 ± 5.24	2.33 ± 5.48	0.018	2.18 (−0.17|4.53)	−2.83 (−6.40|0.74)	0.068	0.118
Breath-holding duration (BHT)	42.22 ± 23.36	61.09 ± 31.60	42.04 ± 18.15	59.35 ± 20.96	37.16 ± 25.50	36.01 ± 19.76	0.000	20.94 (12.65|29.24)	20.34 (7.64|33.04)	0.000	0.002
Breath reflex threshold (BHT-1)	27.34 ± 16.07	41.07 ± 23.18	28.85 ± 19.48	34.30 ± 20.18	20.53 ± 9.68	22.10 ± 10.04	0.001	13.04 (6.22|19.87)	5.85 (−4.47|16.18)	0.000	0.262
Breath reflex suppression (BHT-2)	14.88 ± 14.77	20.02 ± 18.38	13.19 ± 9.30	25.04 ± 25.97	16.57 ± 20.67	13.91 ± 13.95	0.004	7.41 (1.60|13.23)	13.87 (4.91|22.83)	0.013	0.003

AE = Adverse event, CI = Confidence Interval, BAI = Beck Anxiety Inventory, BDI-II = Beck Depression Inventory Revision, BHT = Breath-Holding Scale, DTS = Distress Tolerance Scale, ERQ = Emotion Regulation Scale, ESRQ = Emotional Stress Reaction Questionnaire, *N* = Number of patients, PCL-5 = Post-traumatic Stress Disorder Checklist for DSM-5, PROMIS = patient-reported outcomes measurement information system, PTSD = Post-traumatic stress disorder, SBC = scale of body connection, SF-12 = Short Form 12 Health Survey, TF-CBT = trauma-focused cognitive behavioral therapy.

### 3.4. Analyzes of secondary outcomes

Exploratory ITT analyzes of secondary outcomes revealed no significant post-intervention differences between the pranayama-assisted TF-CBT group and the control group (*p* ≥ 0.050) except for breath-holding duration (*p* < 0.050; Table 4). In PP analyzes, a significant group difference between pranayama patients without AEs and control patients was observed for mental quality of life (*p* = 0.007), while pranayama patients with recurrent AEs reported significantly worsened anxiety (*p* = 0.037) and depression (*p* = 0.039) in comparison to control (Table 5).

### 3.5. Analyzes of pranayama tolerance

**Figure 2:**
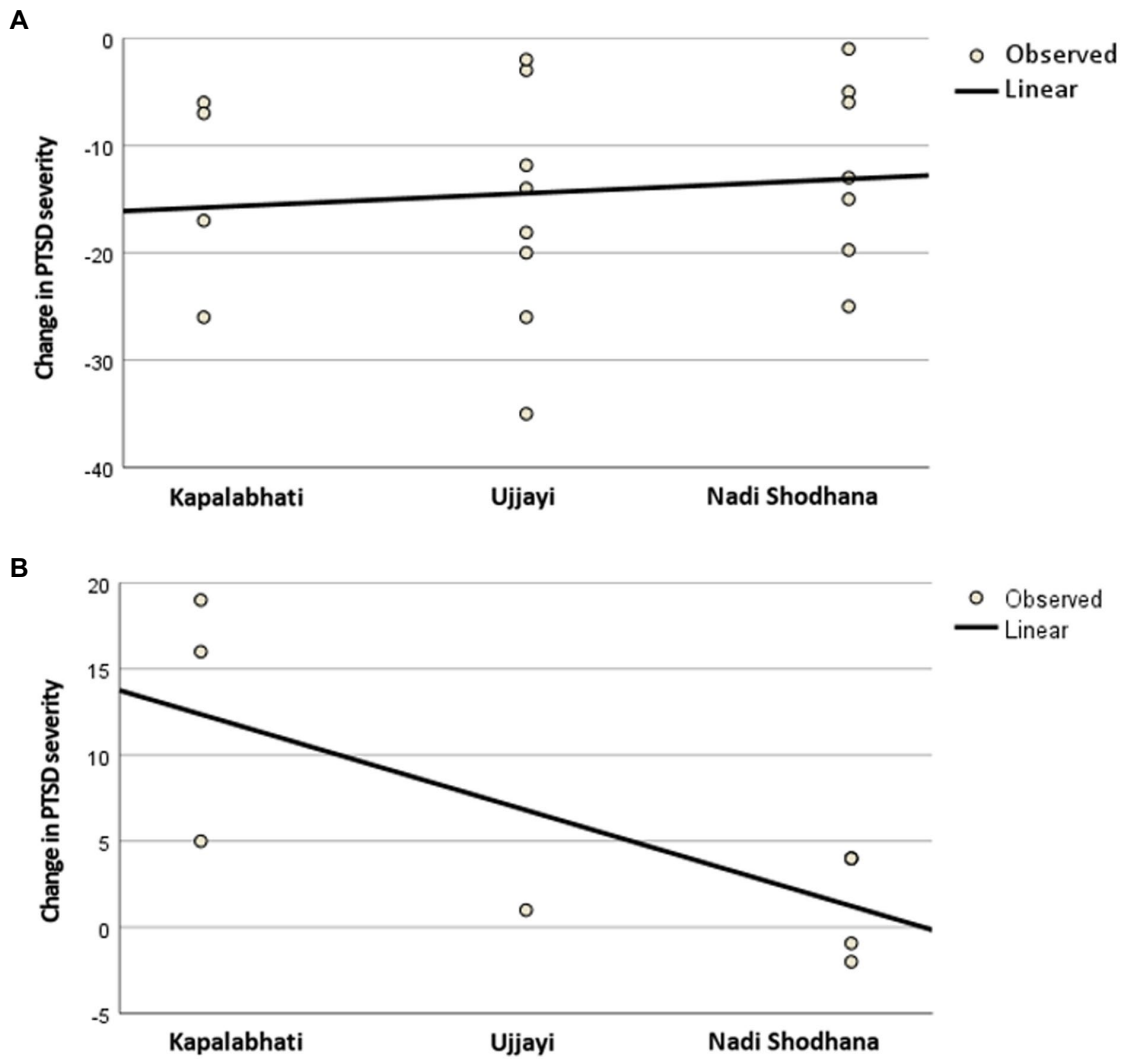
Results of the linear regression analysis for **(A)** patients without recurrent adverse events during pranayama and for **(B)** patients with recurrent adverse events during pranayama. PTSD = Post-traumatic stress disorder.

As shown in Table 3, both pranayama subgroups, those with and without AEs, did not differ significantly in their selection between Kapalabhati, Ujjayi, or Nadi Shodhana (*p* = 0.674). Moreover, performing a special meditative pranayama breathing technique did not cause dropout (*p* = 0.175). However, additional regression analyzes showed that performing Kapalabhati versus Ujjayi or Nadi Shodhana was a significant linear predictor of poorer primary study outcome (worsening in PTSD severity) in the pranayama group with recurrent AEs (B = -5.57; SE = 1.186; *p* = 0.020; adjusted R2 = 0.49). In the pranayama group without AEs, the linear regression was not significant (B = 1.33; SE = 3.01; *p* = 0.664; adjusted *R*^2^ = -0.05; Figure 2).

### 3.6. Moderation analysis

**Figure 3:**
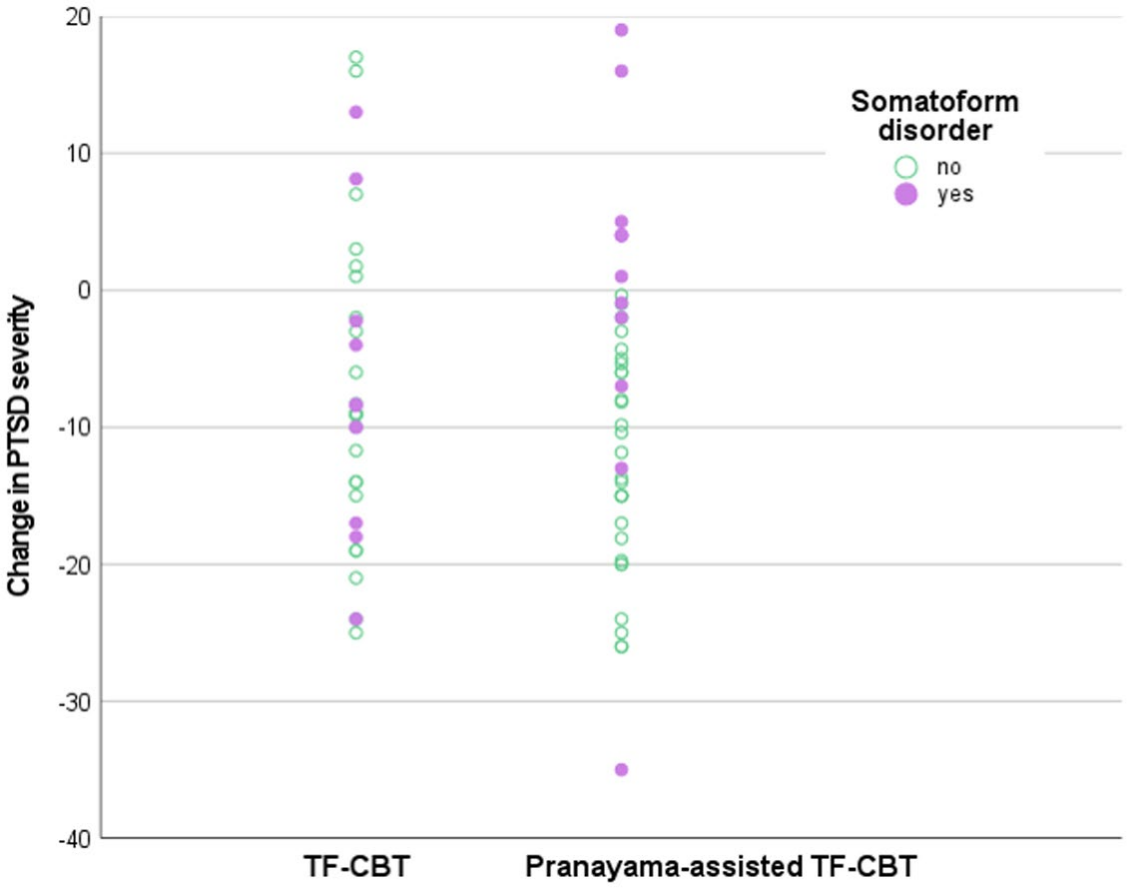
Scatterplot of the moderation analysis. PTSD = Post-traumatic stress disorder, TF-CBT = Trauma-focused cognitive behavioral therapy.

A concurrent diagnosis of an ICD-10 F45 somatoform disorder significantly moderated the effect of treatment allocation on change in PTSD severity with b_3_ = −11.90 (95% CI = -22.56|-1.24; p = 0.029). Descriptive analyzes of the conditional effects of treatment allocation on change in PTSD severity found that patients without a somatoform disorder reported a mean reduction in PCL-5 score of −4.96, while patients with a somatoform disorder reported a worsening of 6.94. The scatterplot (Figure 3) revealed that control patients with somatoform disorders were equally distributed regarding the amount of change in PTSD severity, while pranayama patients with somatoform disorders showed a skewed distribution toward a worsening in PTSD severity. Other tested comorbidities, sociodemographic and disease characteristics (as displayed in Table 2) did not significantly moderate primary study outcome.

## 4. Discussion

### 4.1. Summary of evidence

Pranayama might increase the effects of standard TF-CBT, as long as PTSD patients are able to tolerate the breathing techniques used. In those and only in those cases, 5 to 10 min of pranayama seem to stabilize patients more efficiently than standard stabilization techniques alone leading to lower PTSD severity after 10 TF-CBT sessions. In contrast, if pranayama techniques trigger AEs associated with traumatic memories, pranayama, in particular Kapalabhati and Kumbhaka, appear to destabilize patients and led to worsening of PTSD severity in comparison to TF-CBT alone. In summary, ITT analysis of the primary study outcome did not reveal significant differences.

Reasons for opposing trends in ITT and PP analyzes may be due to the co-occurrence of ICD-10 F45 somatoform disorders in 31% of sample, which moderate the effect of pranayama on PTSD severity. While a diagnosis of concurrent somatoform disorder does not influence primary study outcome in the control group, it increases the probability of experiencing AEs during or subsequent to pranayama and aggravates PTSD severity.

### 4.2. Discussion of the results

The experience of trauma is inextricably linked to the human breath. During trauma exposure, predominantly subcortical survival patterns lead to phylogenetic freezing reflexes associated with bradycardia and flattening of the breath (hypoxia) (54). While initial studies have investigated meditative breathing techniques as a feasible stabilization technique for PTSD (20), breath holding exercises serve as a valid measure of distress tolerance (23).

In our study, we operationalized mindful deep breathing techniques (Kapalabhati, Ujjayi and Nadi Shodhana) to stabilize subcortical ANS reflexes and combined them with an aversive breath holding technique (Kumbhaka) to increase cortical connectivity for more intentional control and the experience of self-efficacy. However, the hypothesis of combining stimuli for stabilization together with targeted exposition does not work for PTSD patients in general.

By practicing Kapalabhati, Ujjayi and Nadi Shodhana, most of the study patients reported a decreased need for breathing, i.e., mild hypoxia (55), which implies heart rate variability (56–58) and thus the ability of the ANS to be responsive to external stimuli. This is only possible during sympathetic and ventral vagal activation, not during dorsal vagal activation (26). In a state, where visceral as well as subcortical and cortical feedback indicate relaxation and safety, targeted attention to respiration and its retention may strengthen the patients’ central executive network, which in turn downregulates amygdala activation (59) and sympathetic arousal (60), despite apparent hypoxia (61).

A subsample of patients with concurrent somatoform disorders reported more severe AEs already during Kapalabhati. Kapalabhati in contrast to Ujjayi and Nadi Shodhana uses fast and forceful, not slow and deep breathing. Both lead to heart rate reliability and ANS adaptability (56–58), Kapalabhati however in combination with rather hyperarousal and sympathetic activation than hypoarousal and ventral-vagal activation. For this reason, highly probably, over the half of the entire sample switched to Ujjayi or Nadi Shodhana. However, a concurrent somatoform disorder is accompanied by more severely altered body and brain structures related to increased hypervigilance and catastrophizing (62, 63). This may explain, why Kapalabhati was associated with worsening in PTSD severity only in the subsample with somatoform disorders. Mild hypoxia during the stabilization exercises and more sustained hypoxia during the breath holding task may cause tissue relaxation as well (64). If tense tissue releases, emotions do likewise (65, 66) and are more probably detected as potentially threatening by patients with somatoform symptoms. Due to their more altered brain structures within the central executive network and their suppressed top-down regulation of, e.g., the amygdala (67), emerging triggers can rarely be tolerated, calmed or re-evaluated. This fosters ongoing amygdala activation, triggers activation patterns of the dorsal vagal loop (26), and often results in AEs, as reported by study patients with concurrent somatoform symptoms.

### 4.3. Strength and limitations

The pragmatic design of the study allows testing the effectiveness and safety of pranayama not as an addition but within the setting of standard, guideline recommended CBT for PTSD. With 20 and 17.6%, patient loss was equally distributed between groups and within the calculated range implying feasibility and tolerability of the study intervention. The pragmatic design increases the generalizability of the results but did not allow concluding, whether pranayama has led to objective alleviation in PTSD or merely subjective responses due to placebo effects. This requires testing within a sham design or a comparison with standard breathing techniques as well as a blinded assessment of PTSD severity. A further pragmatic design would, however, benefit from controlling of unspecific treatment effects such as the quality of the therapeutic alliance (68) as well as from more standardization of trauma-focused CBT, e.g., in terms of the number of performed expositions/confrontation. Further imaging studies investigating changes in functional and structural brain areas during or after pranayama would provide more evidence about the specific effects of pranayama and support the description of possible mechanisms, especially those related to hypoxia (61).

## 5. Conclusion

Clinicians might use pranayama complimentary to standard TF-CBT stabilization techniques. However, study results do not allow drawing final conclusions. Therapists should be aware that in particular breath-holding techniques may be associated with recurrent AEs but do not lead PTSD patients discontinuing the intervention. This can cause worsening of symptoms, especially in PTSD patients with concurrent somatoform disorders. If patients are able to tolerate the yogic breathing and breath-holding techniques, the integration into standard trauma-focused CBT might be worth trying addition to standard stabilization techniques.

## Data availability statement

The raw data supporting the conclusions of this article will be made available by the authors, without undue reservation.

## Ethics statement

The studies involving human participants were reviewed and approved by Ethics committee of the University of Duisburg-Essen, Germany (17-7703-BO). The patients/participants provided their written informed consent to participate in this study.

## Author contributions

HH was responsible for the conception and design of the study, for the analysis and interpretation of the data as well as for writing the article. DM participated in the conception and design of the study, interpretation of the data and writing the article. HC participated in conception, design, and analysis and revised the article critically. All authors contributed to the article and approved the submitted version.

## Conflict of interest

DM was the founder of the intervention and has financial relationships with commercial interests.

The remaining authors declare that the research was conducted in the absence of any commercial or financial relationships that could be construed as a potential conflict of interest.

## Publisher’s note

All claims expressed in this article are solely those of the authors and do not necessarily represent those of their affiliated organizations, or those of the publisher, the editors and the reviewers. Any product that may be evaluated in this article, or claim that may be made by its manufacturer, is not guaranteed or endorsed by the publisher.

## Footnotes

^1^ www.randomisation.com
